# Warming During Different Life Stages has Distinct Impacts on Host Resistance Ecology and Evolution

**DOI:** 10.1111/ele.70087

**Published:** 2025-02-21

**Authors:** Jingdi Li, Cameron A. Smith, Jinlin Chen, Kieran A. Bates, Kayla C. King

**Affiliations:** ^1^ Department of Biology University of Oxford Oxford UK; ^2^ Department of Zoology University of British Columbia Vancouver Canada; ^3^ Blizard Institute, Faculty of Medicine and Dentistry Queen Mary University of London London UK; ^4^ Department of Microbiology & Immunology University of British Columbia Vancouver Canada

**Keywords:** *C. elegans*, host–pathogen interactions, infection, life stages, resistance evolution, warming

## Abstract

Climate change is increasing extreme heating events and the potential for disease outbreaks. Whether hosts can adapt to infection with rising temperatures is important for forecasting species persistence. We tested whether warming—at different host life stages—affects the ecological and evolutionary dynamics of resistance in *Caenorhabditis elegans* infected by a wild bacterial pathogen. We competed resistant and susceptible genotypes across 10 passages and tracked the spread of resistance in the population. Infection and prolonged warming strongly selected for the resistant genotype. Warming during host development induced plastic defences against infection, reducing the selective pressure for costly genetic‐based resistance. Resistance was lost under ambient temperatures and periodic warming. Selection for resistance was likely weakened at ambient temperatures by the dilution effect, whereby the resistant genotype reduced pathogen transmission. Evolutionary dynamics of resistance depend on the balance among pathogen virulence, costs of genetic‐based resistance, the dilution effect and plastic defences induced by temperature stress.

## Graphical abstract

Investigating whether animals can adapt to infection with rising temperatures is important for forecasting their persistence under climate change. Using a nematode host–bacterial pathogen system, our study shows that prolonged warming selects for resistance. The evolutionary dynamics of resistance depended on the balance among pathogen virulence, costs of genetic‐based resistance, the dilution effect, and plastic defences induced by temperature stress.

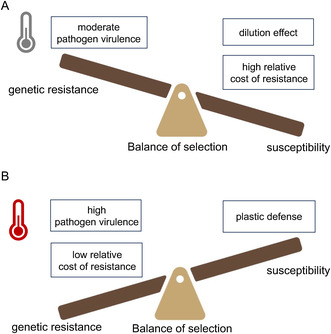

## 1 Introduction

Global climate change is leading to higher average temperatures, as well as more prolonged and extreme heat events (Dosio et al. 2018). Shifting environmental temperatures have been shown to modify host–pathogen dynamics (Rohr et al. 2013; Franke et al. 2019; Kunze et al. 2022; Lafferty and Mordecai 2016), which can worsen infection outcomes for hosts and cause declines in wildlife populations (Jones et al. 2008; Mora et al. 2022).

Warming can impact host–pathogen interactions on ecological timescales. Pathogens can become more virulent (Oh et al. 2009; Kimes et al. 2012; Li et al. 2024a) and transmissible (Shocket et al. 2018) when infecting hosts at warmer temperatures. Hosts can die more during infection if their immune defences are compromised by higher temperatures (Hector et al. 2021, 2023). Alternatively, early exposure to heat stress has shown to plastically enhance defence against infection later in life (Liew et al. 2003; Singh and Aballay 2006; Lee et al. 2012; Prithika et al. 2016; Janda et al. 2019). In *Caenorhabditis elegans* , this plastic defence likely arises from heat stress activating heat shock transcription and protein production, part of the host multipathogen defence pathway (Singh and Aballay 2006; Prithika et al. 2016). Organisms will generally face varying temperatures across their lifetime as climate change brings an increasing frequency and duration of heat periods (IPCC 2013; Zhang et al. 2015). Invertebrates, in particular, are likely to encounter different thermal conditions at developmental and adult stages (Kingsolver et al. 2011). The ability to maintain or evolve resistance to infectious diseases—defined as the ability to limit pathogen burden and lower pathogen‐induced harm (Schneider and Ayres 2008)—is crucial for the persistence of wildlife species as the world warms over time. However, the effects of warming across different host life stages on resistance evolution remain unexplored.

With global mean surface temperatures projected to rise to 4°C by 2100 (Arias et al. 2023), warming will exert multigenerational effects on animals and their interactions with pathogens. Across systems, the environment can shape the strength of selection and specificity in host–pathogen interactions (Wolinska and King 2009). Natural populations often consist of host genotypes that vary in their resistance to infections. Genetic‐based resistance will be strongly favoured in these populations by more virulent infections (Baalen 1998; Wendling et al. 2022) under warming conditions. Host resistance strategies are often found to be costly (Antonovics and Thrall 1994; Bartlett et al. 2018; Graham et al. 2005; Schmid‐Hempel 2003; Schwenke et al. 2016), though with exceptions (Penley et al. 2018). Resistance could be lost over generations if its cost outweighs benefits. For example, the presence of plastic host defences (inheritable and noninheritable) triggered by temperature (Lee et al. 2012; Prithika et al. 2016; Janda et al. 2019; Baugh and Day 2020) might reduce the selective benefits of genetic‐based resistance. The reduction in benefits could also occur by the presence of ‘dilution effect’ (Keesing and Ostfeld 2021; Civitello et al. 2015), as the resistant genotype in a population can benefit susceptible individuals in populations by reducing pathogen density and transmission in the environment (Keesing and Ostfeld 2021; Civitello et al. 2015). It is unclear whether warming can shift the relative costs and benefits of resistant genotypes, with consequences for the dilution effect.

Here, we directly tested the above hypotheses using *Caenorhabditis elegans* and a bacterial pathogen *Leucobacter musarum*, naturally isolated from congener nematodes in rotting banana stems in Cape Verde (Hodgkin et al. 2013). This pathogen is novel to *C. elegans* but can adhere to the cuticle of the lab‐adapted N2 strain, causing lethal infection. The mutant *srf‐2* is resistant to *L. musarum* as it has altered surface antigenicity preventing pathogen attachment (Hodgkin et al. 2013). Using this experimental system, we investigated the impact of warming across host life stages on resistance to a novel pathogen, across both ecological and evolutionary timescales. We compared fitness metrics (mortality and fecundity) of susceptible and resistant genotypes maintained alone or in mixed populations in response to prolonged or periodic warming, as well as ambient regimes. We then conducted an evolution experiment, tracking the frequency of the resistant genotype in host populations across 10 generations, in the presence or absence of pathogens. We further developed a mechanistic model to disentangle the impact of different environmental and host factors on the evolutionary dynamics of resistance.

## 2 Materials and Methods

### 2.1 The Nematode‐Pathogen System

Both N2 and *srf‐2* worm genotypes were originally obtained from the Caenorhabditis Genetics Centre (CGC, Minnesota, USA). They have similar generation times (O'Rourke et al. 2023). The *srf‐2* resistant strain has a green florescent reporter and can be distinguished from the unlabeled susceptible genotype. This GFP insertion does not carry a host fitness cost (Hodgkin et al. 2001). *Leucobacter musarum* was obtained from Hodgkin laboratory (Hodgkin et al. 2013). This genus has been shown to naturally infect *C. elegans* (Bates and King 2021; Bates et al. 2021). The environmental growth of this pathogen on NGM agar (a less nutrient‐rich media specially designed to support *C. elegans*) at 20°C or 25°C is likely similarly limited over the 24‐h infection exposure period. The pathogen grows relatively slowly in vitro, taking approx. 48 h to form colonies on LB agar at 30°C. Nematode and bacterial maintenance were performed following protocols in Li et al. (2024b).

### 2.2 Nematode Population Survival and Fecundity

We manipulated temperature (ambient vs. elevated) and its timing (periodic vs. prolonged) during worm development (from L1 larvae to L4 young adults) and during pathogen exposure at adult stage. We used the ambient temperature of 20°C and a warmer temperature of 25°C. The temperature regimes are ambient (20°C–20°C), periodic warming (20°C–25°C, 25°C–20°C) and prolonged warming (25°C–25°C). An ambient 20°C is standard for maintaining *C. elegans* in laboratory and is optimal for nematode reproduction (Gouvêa et al. 2015). Warming at 25°C causes mild heat stress in *C. elegans* . This temperature shortens their lifespan, reduces reproductive output (Xiao et al. 2013; Gouvêa et al. 2015), and accelerates development (Sekajova et al. 2022). Exposing nematodes at 25°C can result in higher *L. musarum* virulence, as measured by host mortality (Hodgkin et al. 2013; Bates et al. 2021).

For the pathogen exposure assay, susceptible or resistant L1 larvae were grown on *E. coli* OP50 food at either 20°C or 25°C for ~48 h or ~ 36 h, respectively, until they reached stage L4. L4 young adults were washed off the plate, gravity washed twice and transferred onto infection or control plates, and left for 24 h at either 20°C or 25°C. We transferred approx. 300–400 L4‐stage worms onto each 5.5 NGM plate. Transferred worm populations were either 100% susceptible or resistant, or mixed populations consisted of 50% susceptible and 50% resistant worms. Each treatment was replicated six times.

Infected host mortality and fecundity were assessed. The number of live and dead nematodes were counted on each plate after pathogen exposure. Nematodes killed by the pathogen typically showed straight and stiff appearance. They were confirmed dead when they did not respond to touch with a platinum wire. To assess population fecundity, all worms and eggs were washed off the plate, and unhatched sterile eggs were collected by bleaching. We sampled for offspring slightly earlier in warming treatment to compensate for the potential age difference that induced earlier reproduction. After 12 h incubation in M9 buffer, numbers of the total and resistant L1 larvae were counted in six 5 uL drops under a fluorescent microscope (40× magnification).

### 2.3 Measuring Pathogen Colonisation in Host Genotypes

To confirm resistance in the *sfr‐2* mutant, we measured pathogen load in both genotypes using a colony‐forming unit (CFU) assay. Similarly, 300–400 L4‐stage worms (50%:50% susceptible: resistant) were added to pathogen exposure or control NGM plates (replicated four times) for 24 h at 25°C. To quantify pathogen colonisation, five worms of each genotype were picked from each plate and transferred to a tryptic soy broth (TSB) agar plate to which 1000uL of M9 media had been suspended on the surface of the agar. Worms were washed in M9 for 10 min and then transferred to a bead beating tube containing 1 mL of M9. Tubes were placed in a bead beater for 2 min and then centrifuged at 3500 rpm for 1 min. The supernatant was diluted and spread onto a 9 cm LB agar plate and incubated at 25°C, then CFUs were counted.

### 2.4 Evolution Experiment

**FIGURE 1:**
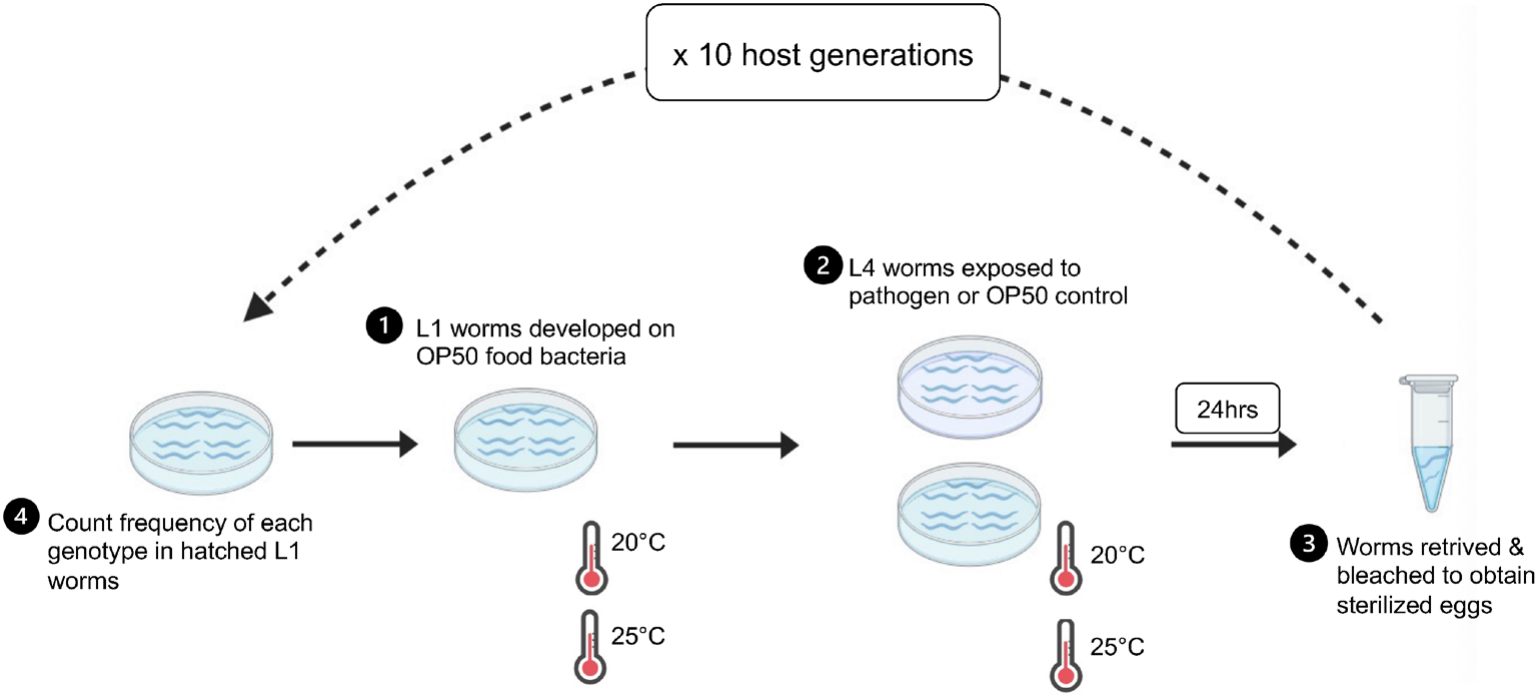
Schematic illustration of the evolution experiment competing host genotypes. In brief, L1‐stage host population consisted of susceptible N2 and resistant *srf‐2* were grown on *E. coli* OP50 food bacteria at 20°C or 25°C until L4 stage. L4 worms were exposed to pathogen or food control at 20°C or 25°C. After 24 h, worm populations were bleached to obtain sterilised eggs. The frequency of each genotype was determined in the hatched L1 stage. All hatched L1 worms were grown to L4 and returned to the corresponding infection and temperature treatment. Ten passages were made.

Following the same protocol in single‐generation experiments, we competed the susceptible N2 and the resistant *srf‐2* genotypes, and we tracked their frequency dynamics under different infection and temperature regimes (Figure 1). The experiment started with approx. 500 nematodes of each genotype per replicate. Population sizes were consistently maintained at approx. 1000 nematodes per passage. All treatments and controls were replicated six times. At the end of each passage, the number of hatched L1 worms per uL was used to estimate the volume required to obtain approximately 1000 L1 worms. L1 worms from each replicate were placed onto OP50 food plates and raised to L4 stage before being placed back into treatments or controls. For several replicates where there were < 1000 worms, individuals were added from either 20°C or 25°C uninfected stock plates to keep population sizes constant across replicates. To account for this addition possibly weakening epigenetic resistance (if any) for replicates in infection treatment, the evolutionary frequency data were analysed with and without these replicates.

Genotype frequency was calculated by subsampling. A genotype was considered lost if it was not observed for two consecutive generations. The replicate population was no longer passaged if loss was observed.

### 2.5 Statistical Analyses

Unless specified, all analyses were conducted in R 4.1.0 (RStudio 2023.03.1 + 446). Pathogen CFUs counts were compared across host genotypes using a *t*‐test. Host mortality data were fitted in generalised linear model (GLM) with quasibinomial distribution. We stratified host types to four different groups: S‐alone (susceptible hosts maintained alone), S‐mixed (susceptible hosts in mixed populations), R‐alone (resistant hosts maintained alone) and R‐mixed (resistant hosts in mixed populations). As the number of susceptible individuals in mixed population is half the number of susceptible‐only population, we scaled the mortality of susceptible‐mixed group by doubling the original host mortality. Fecundity data were fitted in GLM with negative binomial distribution. Pairwise comparison tests based on GLM model were conducted using emmeans function in emmeans R library.

The proportion of the resistant genotype among offspring was fitted in GLM with binomial distribution (with total number of offspring as weights in model). Model performance was evaluated by QQ‐plot and dispersion test to ensure they addressed the overdispersion of the data. To test which genotype dominated in the offspring population under different treatments, we used exact binomial tests on each replicate. *p* values were pooled to obtain group‐level significance using poolr R package (Cinar and Viechtbauer 2022). Details of all statistical results are in Table S5.

For the evolution experiment, we calculated the relative fitness of the resistant genotype compared with the susceptible genotype. Resistance relative fitness was calculated as a function of the change in genotype frequencies using the equation ln (*f* resistant/*f* susceptible) where *f* represents genotype frequencies (Bates et al. 2021). In cases where one genotype had a frequency of zero, we assumed a frequency of 1/(*N* + 1) for this genotype (*N* is population size). Mean and sd of resistance relative fitness across 10 generations were summarised. We used the Wilcoxon rank sum test to test for differences in mean relative fitness between treatment groups. To evaluate the trend of genotype frequency change across host generations under different treatments, we fitted the frequency data using a generalised linear mixed model (GLMM) with a binomial distribution, as well as a Bayesian model with a binomial distribution. As results were consistent between both approaches, only the GLMM modelling details are shown. Genotype dominance at generation ten was assessed using binom.test and poolr R package. Details of all statistical results are in Table S6. The assumptions, simulations and results of the mechanistic model are in Supporting Informations.

## 3 Results

### 3.1 Impacts of Warming and Infection on Genotype Fitness

**FIGURE 2:**
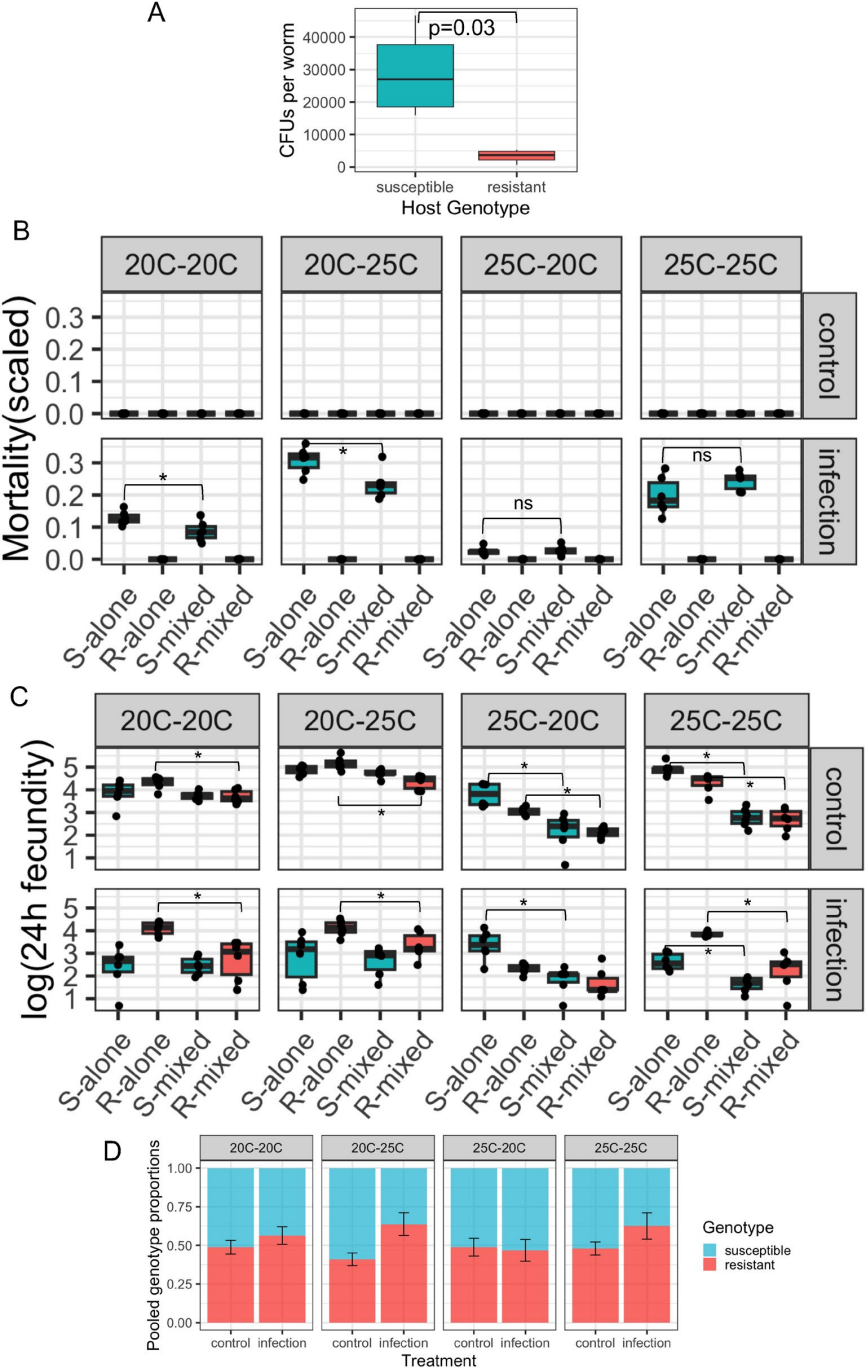
Pathogen load, mortality and fecundity of susceptible (S) and resistant (R) host genotypes in ecological time experiments. (A) Pathogen load, measured by number of CFUs per host individual. (B) Proportion of S and R hosts killed from pathogen infection, alone or in mixed populations. Mortality in mixed populations was scaled (by doubling). (C) Fecundity of S and R genotypes, alone or in mixed populations. (D) Proportion of S and R genotypes in total offspring from mixed populations. Proportions are pooled from six replicates in each treatment group. Error bars represent SE for the proportion of resistant genotype. Significant difference in pairwise comparison is indicated by an asterisk.

We found that pathogen load was significantly higher in the susceptible (median CFUs per worm = 27,000) than in the resistant genotype (median CFUs per worm = 3667) (Table S2, Figure 2A, *t*‐test: *t*_(3.13)_ = 3.59, *p* = 0.03). In single‐generation experiments, significant mortality was observed for susceptible hosts under infection, while no mortality was observed in the resistant populations exposed to *L. musarum* (Table S3, Figure 2B). No mortality was found in uninfected controls.

Warming at different life stages had a distinct impact on infection mortality. For susceptible hosts, warming during host development reduced infection‐induced mortality (25°C vs. 20°C es = −1.73, *p* < 0.001), while warming during adult‐stage infection caused higher mortality (Hodgkin et al. 2013) (25°C vs. 20°C es = 1.11, *p* < 0.001). Notably, at 25°C–25°C, where developmental warming reduced mortality and infection warming increased mortality, host mortality was higher than in 20°C–20°C. In this latter treatment, there was no warming‐mediated effect (25°C–25°C vs. 20°C–20°C: es = 0.52, *p* = 0.002).

Susceptible hosts were protected by the resistant genotype in mixed populations. Susceptible hosts in mixed populations exhibited significantly lower mortality from infection, compared with when they were alone (Figure 2B, 20°C–20°C: mixed vs. alone es = −0.43, *p* = 0.021; 20°C–25°C: mixed vs. alone es = −0.37, *p* = 0.004). This result suggests that the presence of the resistant genotype may have conferred protection against infection, potentially by diluting pathogens from the environment (Keesing et al. 2006). This dilution effect was not observed when hosts developed at warmer temperatures, where similar mortality levels were observed between susceptible hosts alone and those in mixed populations (Figure 2B, susceptible alone vs. mixed at 25°C–20°C: *p* = 0.811; 25°C–25°C: *p* = 0.052).

We observed a significant competitive or relative cost for the resistant genotype, regardless of pathogen exposure. In mixed populations without pathogen exposure, we found that resistant hosts had a significant lower fecundity compared with when they were alone, across all temperature regimes (Figure 2C, resistant mixed vs. alone: es = −0.61, *p* < 0.001). In contrast, susceptible hosts only had reduced fecundity in mixed populations during developmental warming regimes (Figure 2C). When exposed to the pathogen, infection reduced the fecundity of both genotypes, with the effect varying across temperature regimes (Figure 2C). Infection significantly decreased susceptible host fecundity across most temperature regimes, except in 25°C–20°C (Table S5, *p* < 0.0001), where the lowest infection‐induced mortality was observed. When maintained alone, resistant hosts produced significantly more offspring than susceptible hosts under infection, across all temperature regimes except in 25°C–20°C (Figure 2C). This fecundity advantage disappeared in mixed populations, where both genotypes showed similar fecundity levels (Figure 2C), suggesting a significant relative cost for resistance. Notably, resistant hosts consistently had the lowest fecundity in the 25°C–20°C regime, regardless of pathogen exposure or population type.

We found that infection significantly increased the proportion of the resistant genotype in total offspring, compared to uninfected controls (Figure 2D, infection es = 0.76, *p* < 2e‐16). This effect was exaggerated by warming during infection (infection: 25°C es = 0.64, *p* = 0.0007). The resistant genotype dominated the offspring population under infection across most temperature regimes, except in 25°C–20°C (Figure 2D, pooled *p* value = 0.002 for 20°C–20°C, 20°C–25°C and 25°C–25°C regimes). As expected, in uninfected treatments, susceptible offspring were in higher proportions (Figure 2D). The 20°C–25°C regime reduced the proportion of resistant offspring most significantly (es = −0.33, *p* = 0.002). However, when combined with infection, the same regime (20°C–25°C) increased the proportion of resistant offspring to the greatest extent (20°C–25°C:infection es = 0.54, *p* = 0.015).

### 3.2 Evolutionary Dynamics of Host Resistance

**FIGURE 3:**
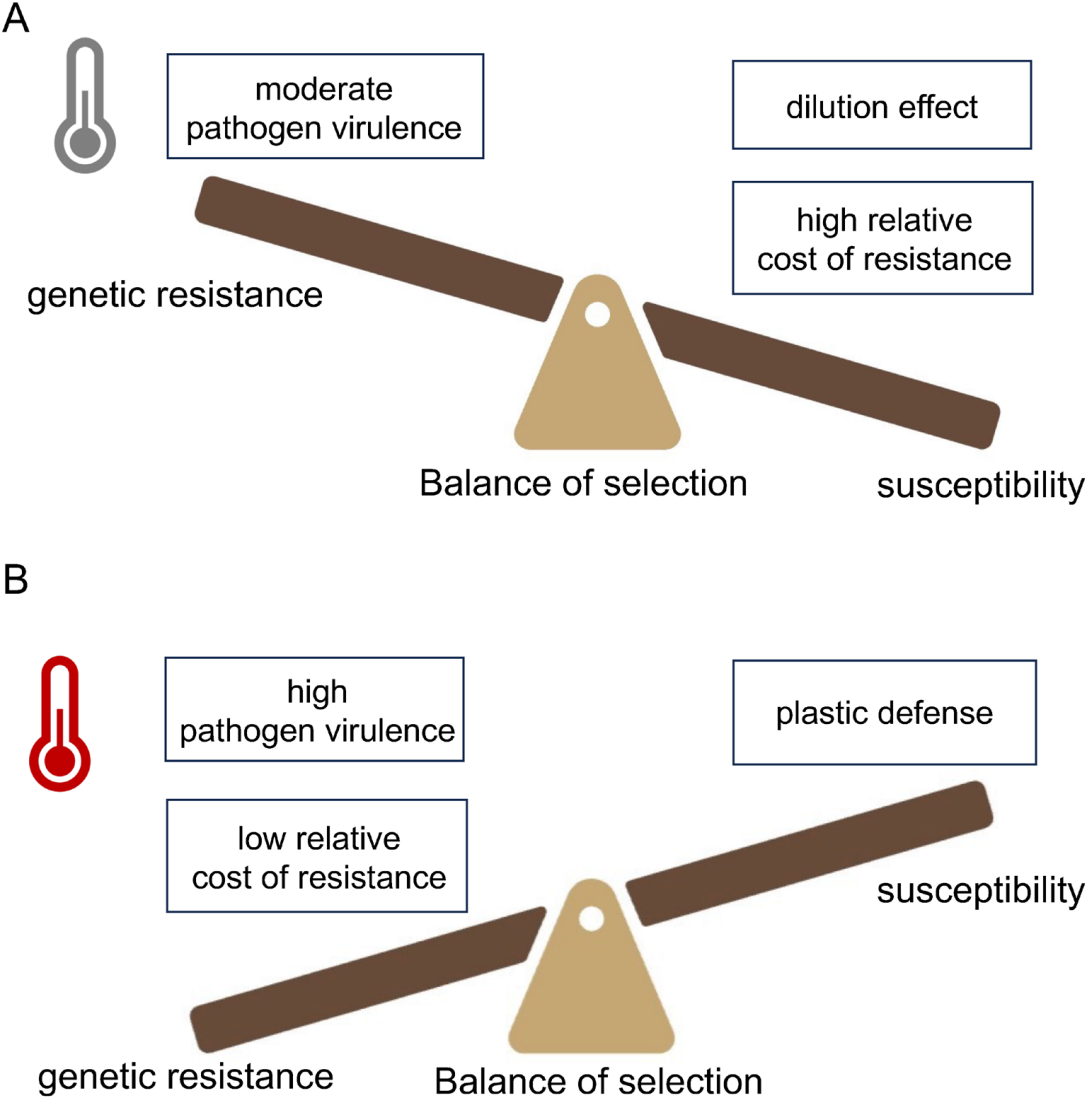
Schematic of predictive framework illustrating the balance of selective forces influencing host resistance evolution. (A) Under ambient temperature conditions, the dilution effect and the relative cost of genetic resistance may outweigh the selective pressure exerted by pathogens with moderate virulence. (B) Under warming conditions, increased pathogen virulence amplifies the benefits of resistance and may select for genetic resistance in the host population. Created with BioRender.

Based on the above ecological‐time outcomes, we developed predictions for host resistance evolution to account for the complexity of our experimental design (Figure 3, summary of ecological outcomes in Table S1). We predicted that in the absence of pathogen or when pathogen virulence was moderate, the resistant genotype would likely be selected against due to the relative cost (Figure 3A). When pathogen virulence was enhanced, however, selection for resistance should be more direct (Figure 3B). We hypothesized that warming during infection would strongly select for the resistant genotype, while the dilution effect and temperature‐mediated plastic defences could reduce the selection for genetic resistance. The evolutionary trajectory of resistance will ultimately depend on the balance between these opposing selective forces, determining whether the resistant genotype is favoured in the long run.

**TABLE 1:** Summary of GLMM, modelling resistance frequency across generations and treatments.

	Estimate	SE	*p*
Main effects			
Generation	−0.21	0.016	< 0.001***
Infection	−0.43	0.346	0.209
20°C–25°C	0.28	0.33	0.399
25°C–20°C	−0.13	0.339	0.691
25°C–25°C	0.39	0.329	0.238
Interaction effects			
Generation: Infection	0.13	0.025	< 0.001***
Generation: 20°C–25°C	0.19	0.019	< 0.001***
Generation: 25°C–20°C	−0.08	0.029	0.005**
Generation: 25°C–25°C	0.16	0.018	< 0.001***
Infection: 20°C–25°C	0.8	0.476	0.091
Infection: 25°C–20°C	0.92	0.49	0.059
Infection: 25°C–25°C	−0.08	0.479	0.867
Generation: Infection: 20°C–25°C	−0.21	0.029	< 0.001***
Generation: Infection: 25°C–20°C	−0.1	0.044	0.025*
Generation: Infection: 25°C–25°C	0.09	0.03	0.003**

*Note:* “*”: *p* ≤ 0.05; “**”: *p* ≤ 0.01; “***”: *p* ≤ 0.001.

**FIGURE 4:**
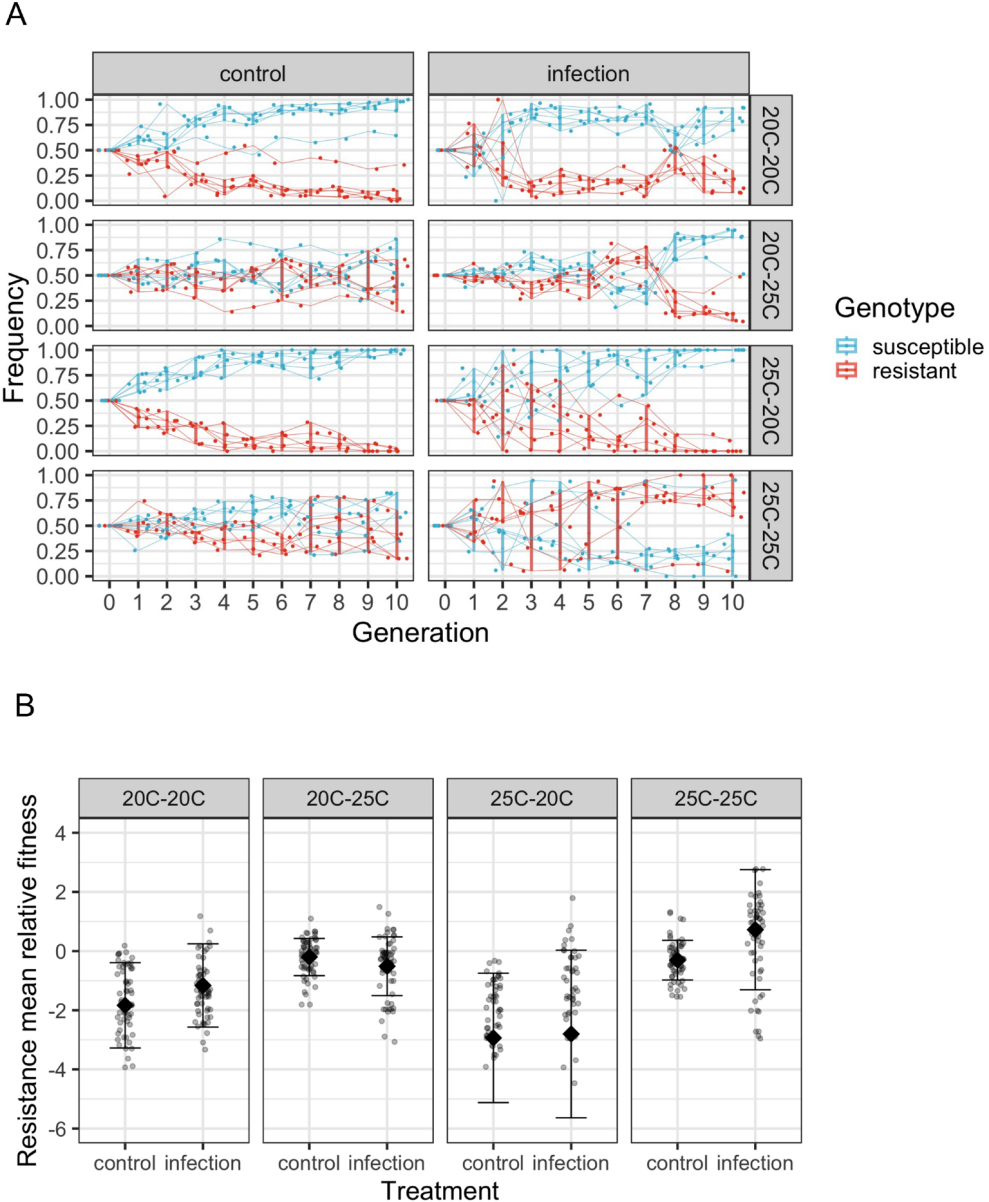
(A) Genotype frequency dynamics for 10 host generations across infection and temperature regimes. Host populations were started at 50:50 ratio of the two genotypes. The resistant genotype was selected in host populations following pathogen exposure and prolonged warming across life stages, while resistance was lost with ambient temperatures and periodic warming. (B) Mean relative fitness of resistant genotype for 10 generations. In each facetted plot, the diamond shape represents mean, error bar represents mean ± SD, each point represents relative fitness of resistant genotype in one replicate.

We found that resistance was regularly lost across passages (Tables 1and S4, GLMM model, generation es = −0.21, *p* < 0.001). This outcome was most likely in uninfected controls, which reveals an evolutionary cost to the resistant genotype (similarly found in Bates et al. 2021). Resistance was lost first in a host population at passage six in the developmental warming regime (25°C–20°C) (Figure 4A, Figure S1). After nine host passages, the resistant genotype was gone in most replicates in the 25°C–20°C regime, regardless of infection (Figure S1). The resistant genotype fixed in only one population in the prolonged warming regime (25°C–25°C) with infection after eight passages (Figure S1).

We found that the presence of the pathogen across generations generally favoured higher levels of resistance (es = 0.13, *p* < 0.001). Prolonged warming increased the frequency of resistance in host populations, compared with ambient temperatures (es = 0.16, *p* < 0.001). This pattern was more pronounced during pathogen infection (es = 0.089, *p* = 0.003). Conversely, when temperature was cooled to 20°C after developmental warming (25°C–20°C), we observed a decrease in resistance frequency across time (es = −0.082, *p* = 0.005), especially combined with infection (es = −0.098, *p* = 0.025). The reverse switch, 20°C–25°C, drove a rapid loss of resistance under infection (es = −0.21, *p* < 0.001). At the end of the evolution experiment, the resistant genotype dominated under prolonged warming (25°C–25°C) with pathogen exposure (frequency > 0.5, pooled *p* value < 0.001), while the susceptible genotype dominated in other regimes (frequency > 0.5, pooled *p* value < 0.033 for all other treatments). These patterns were not altered when we excluded replicates where worms from uninfected stock plates were added (Figure S3).

Pathogen exposure significantly increased the relative fitness of resistance under prolonged warming (Figure 4B. Wilcoxon rank sum test, *p* = 0.003), though this selective advantage was only observed in later generations (Figure S2, Generation 1–5: *p* > 0.222; Generation 6–10: *p* = 0.008). We found that adult‐stage warming increased the relative fitness of the resistant genotype, regardless of pathogen exposure (Figure 4B. control: *p* < 0.001, infection: *p* < 0.001). This trend was significant throughout the evolution experiment (Figure S2. Generation 1–5: control: *p* < 0.001, infection: *p* = 0.012. Generation 6–10: control: *p* < 0.001, infection: *p* = 0.002).

The susceptible genotype persisted or even dominated under infection, at ambient developmental temperature regimes, despite lacking the plastic defences mediated by developmental warming. We hypothesise that the dilution of pathogen cells by the resistant genotype may have helped maintain the abundance of susceptible individuals, which was only detected at ambient developmental temperatures. Our model simulation further supported that the dilution effect could disproportionally benefit the susceptible genotype and played an important role in its persistence (SI text, Figures S4–S6).

## 4 Discussion

Global warming is increasing average temperatures and causing thermal fluctuations, which may escalate disease threats to biodiversity (Rohr et al. 2013; Kunze et al. 2022; Altizer et al. 2003). Pathogens can impose strong selection for host resistance (Koskella 2018), and warming could amplify these effects by increasing host harm from infection (Kimes et al. 2012; Oh et al. 2009; Li et al. 2024b). Our study found that resistance to a novel pathogen in populations was strongly favoured by pathogen presence when warming was prolonged. This result suggests that continuous global warming could enhance animal resistance to emerging infectious diseases.

Resistance was otherwise maintained at lower frequencies or lost in infected host populations experiencing periodic warming or ambient temperatures. Our ecological‐time experiment revealed a significant relative cost of resistance across all treatments. It is well‐documented that resistance to disease often comes with trade‐offs, including costs to host growth (van der Most et al. 2011; Brown and Rant 2013), fecundity (Fuxa and Richter 1998; Webster and Woolhouse 1999) and foraging rates (Hall et al. 2010, 2012; Auld et al. 2013; Kraaijeveld and Godfray 1997). In our system, changes in the cuticle structure of the resistant genotype may impact locomotory efficiency, and consequently the ability to forage or behaviourally avoid pathogens. Over evolutionary time, these costs can result in selection against resistance (Duncan et al. 2011). Interestingly, when warming was periodic (specifically, in the 20°C–25°C regime), the loss of resistance was more pronounced under infection than in uninfected controls. This finding aligns with patterns observed in zooplankton populations, where lower resistance evolved during epidemics compared with nonepidemic periods (Strauss et al. 2017). One explanation is that during epidemics, increased resource availability amplifies the cost of reduced foraging efficiency, outweighing the benefits of resistance (Walsman et al. 2023). In our study, the higher pathogen‐induced mortality of susceptible hosts during adulthood warming may have similarly increased environmental food availability, thereby elevating the relative cost of resistance.

In addition to the relative cost of genetic‐based resistance, plastic changes in host defence induced by thermal stress, may have driven the loss of resistance in our experiment. We found that warming during the susceptible host larval stages reduced pathogen‐induced mortality. In *C. elegans* , environmental stress or pathogen exposure can induce nongenetic heritable and noninheritable host resistance, via histone modifications and small RNA regulation in progeny gene expression (Moore et al. 2019; Palominos et al. 2017; Burton et al. 2020; Wibisono and Sun 2023; Legüe et al. 2022; Baugh and Day 2020; Ghildiyal and Zamore 2009; Fire et al. 1998), or heat shock protein transcription activation (Singh and Aballay 2006; Prithika et al. 2016). Heat exposure at early life stages has shown protective effects across a range of organisms, from broiler chickens to plants ( *Arabidopsis thaliana* ) (Liew et al. 2003; Janda et al. 2019; Lee et al. 2012). We found that whilst the plastic response also carried some cost for the susceptible genotype, it was more advantageous than genetic‐based resistance over evolutionary time. This benefit was offset by heightened pathogen virulence under warmer temperatures. Pathogen virulence is thus indeed a primary driver of host resistance (Boots and Bowers 1999). Hosts can however reciprocally impose selection on pathogen virulence (Mikonranta et al. 2015; Cressler et al. 2016; Brown et al. 2012). The presence of transgenerational virulence (McIntirea et al. 2024) may further complicate the evolutionary outcomes of host resistance. Understanding to what extent warming can thus alter those coevolutionary selection dynamics could provide insights into the future burdens of infectious diseases under climate change (Brooks and Boeger 2019; Wolinska and King 2009; Claar and Wood 2020).

Our study revealed that the dilution effect may have maintained susceptibility in host populations during periodic warming and ambient temperatures. In our system, upon infection, susceptible individuals became heavily colonised by the pathogen, potentially contributing to more pathogen transmission and shedding into the environment. In contrast, resistant hosts carried a lower pathogen load, limiting their potential for shedding or transmission. We used a substitutive design whereby host populations were maintained a constant size and adding resistant hosts reduced the density of susceptible individuals. This setup likely facilitated the observation of dilution effect (Johnson et al. 2015, 2024). By further keeping pathogen density constant across host generations, we minimised confounding effects from fluctuating pathogen densities, making it easier to detect the impact of dilution effect on resistance evolution. This role of this phenomenon in preventing the spread of resistance was further supported by our modelling simulations. At an ecosystem level, the dilution effect may limit disease spread, and biodiversity loss might amplify epidemics, threatening both wildlife and human populations (Civitello et al. 2015). This diversity‐disease relationship is also influenced by environmental factors such as warming (Liu et al. 2016), with effects depending on host life stage during heat exposure (Zhang et al. 2015). Thus, climate‐induced biodiversity loss might further amplify warming‐induced disease susceptibility, worsening the outlook for plant and animal species (Wudu et al. 2023).

In conclusion, we showed that both ecological and evolutionary pathways of host resistance can be shaped by warming, alongside fitness constraints associated with thermal stress. We suggest that selection for resistance in animal populations will be strengthened by prolonged warming climate if pathogen virulence is enhanced. Depending on host life stage at exposure, warming can also mediate protection from infection—thereby reducing selection for genetic‐based resistance. The interplay between the dilution effect and inter‐genotypic competition can outweigh pathogen‐mediated selection, preventing the spread of resistance during epidemics. Understanding these dynamics is essential for forecasting persistence and the health of host populations in an infectious and warming world (Jones et al. 2008; Dosio et al. 2018; Mora et al. 2022).

## Author Contributions

J.L. and K.C.K. conceived and designed the study. J.L. conducted the experiment, and J.L. and K.A.B. collected data, with guidance from K.C.K. J.L., C.A.S. and J.C. conceived the mathematical model. J.L. and C.A.S. ran the simulations. J.L. and K.C.K. wrote the manuscript. All authors contributed to reviewing and editing.

## Conflicts of Interest

The authors declare no conflicts of interest.

### Peer Review

The peer review history for this article is available at https://www.webofscience.com/api/gateway/wos/peer‐review/10.1111/ele.70087.

## Supporting information


Appendix S1.

Tables S1–S6.

## Data Availability

The data and codes that support the findings of this study are openly available in figshare at https://doi.org/10.6084/m9.figshare.28163432.

## References

[ele70087-bib-0001] Altizer, S. , D. Harvell , and E. Friedle . 2003. “Rapid Evolutionary Dynamics and Disease Threats to Biodiversity.” Trends in Ecology & Evolution 18, no. 11: 589–596. 10.1016/j.tree.2003.08.013.

[ele70087-bib-0093] Antonovics, J. , and P. H. Thrall . 1994. “The cost of resistance and the maintenance of genetic polymorphism in host—pathogen systems.” Proceedings of the Royal Society of London. Series B: Biological Sciences 257, no. 1349: 105–110. 10.1098/rspb.1994.0101.

[ele70087-bib-0094] Arias, P. A. , N. Bellouin , E. Coppola , et al. 2023. “Technical Summary.” In Climate Change 2021 – The Physical Science Basis: Working Group I Contribution to the Sixth Assessment Report of the Intergovernmental Panel on Climate Change, 33–144. Cambridge University Press. 10.1017/9781009157896.002.

[ele70087-bib-0003] Auld, S. K. J. R. , R. M. Penczykowski , J. H. Ochs , D. C. Grippi , S. R. Hall , and M. A. Duffy . 2013. “Variation in Costs of Parasite Resistance Among Natural Host Populations.” Journal of Evolutionary Biology 26, no. 11: 2479–2486. 10.1111/jeb.12243. 24118613

[ele70087-bib-0004] van Baalen, M. 1998. “Coevolution of Recovery Ability and Virulence.” Proceedings of the Royal Society B 265: 317–325. 10.1098/rspb.1998.0298. 9523434 PMC1688890

[ele70087-bib-0005] Bartlett, L. J. , L. Wilfert , and M. Boots . 2018. “A Genotypic Trade‐Off Between Constitutive Resistance to Viral Infection and Host Growth Rate.” Evolution 72, no. 12: 2749–2757. 10.1111/evo.13623. 30298913 PMC6492093

[ele70087-bib-0006] Bates, K. A. , J. S. Bolton , and K. C. King . 2021. “A Globally Ubiquitous Symbiont Can Drive Experimental Host Evolution.” Molecular Ecology 30, no. 15: 3882–3892. 10.1111/mec.15998. 34037279

[ele70087-bib-0007] Bates, K. A. , and K. C. King . 2021. “Leucobacter.” Trends in Microbiology 29, no. 11: 1046–1047. 10.1016/j.tim.2021.06.010. 34304971

[ele70087-bib-0008] Baugh, L. R. , and T. Day . 2020. “Nongenetic Inheritance and Multigenerational Plasticity in the Nematode *C. elegans* .” eLife 9: e58498. 10.7554/eLife.58498. 32840479 PMC7447421

[ele70087-bib-0010] Boots, M. , and R. G. Bowers . 1999. “Three Mechanisms of Host Resistance to Microparasites—Avoidance, Recovery and Tolerance—Show Different Evolutionary Dynamics.” Journal of Theoretical Biology 201, no. 1: 13–23. 10.1006/jtbi.1999.1009. 10534432

[ele70087-bib-0012] Brooks, D. R. , and W. A. Boeger . 2019. “Climate Change and Emerging Infectious Diseases: Evolutionary Complexity in Action.” Current Opinion in Systems Biology 13: 75–81.

[ele70087-bib-0013] Brown, J. K. M. , and J. C. Rant . 2013. “Fitness Costs and Trade‐Offs of Disease Resistance and Their Consequences for Breeding Arable Crops.” Plant Pathology 62, no. S1: 83–95. 10.1111/ppa.12163.

[ele70087-bib-0014] Brown, S. P. , D. M. Cornforth , and N. Mideo . 2012. “Evolution of Virulence in Opportunistic Pathogens: Generalism, Plasticity, and Control.” Trends in Microbiology 20, no. 7: 336–342. 10.1016/j.tim.2012.04.005. 22564248 PMC3491314

[ele70087-bib-0015] Burton, N. O. , C. Riccio , A. Dallaire , et al. 2020. “Cysteine Synthases CYSL‐1 and CYSL‐2 Mediate *C. elegans* Heritable Adaptation to *P. vranovensis* Infection.” Nature Communications 11, no. 1: 1741. 10.1038/s41467-020-15555-8. PMC7142082 32269224

[ele70087-bib-0017] Cinar, O. , and W. Viechtbauer . 2022. “The Poolr Package for Combining Independent and Dependent p Values.” Journal of Statistical Software 101, no. 1: 1–42. 10.18637/jss.v101.i01.

[ele70087-bib-0018] Civitello, D. J. , J. Cohen , H. Fatima , et al. 2015. “Biodiversity Inhibits Parasites: Broad Evidence for the Dilution Effect.” Proceedings of the National Academy of Sciences 112: 8667–8671. 10.1073/pnas.1506279112. PMC4507196 26069208

[ele70087-bib-0019] Claar, D. C. , and C. L. Wood . 2020. “Pulse Heat Stress and Parasitism in a Warming World.” Trends in Ecology & Evolution 35, no. 8: 704–715. 10.1016/j.tree.2020.04.002. 32439076

[ele70087-bib-0020] Cressler, C. E. , D. V. Mcleod , C. Rozins , J. Van Den Hoogen , and T. Day . 2016. “The Adaptive Evolution of Virulence: A Review of Theoretical Predictions and Empirical Tests.” Parasitology 143, no. 7: 915–930. 10.1017/S003118201500092X. 26302775 PMC4873896

[ele70087-bib-0022] Dosio, A. , L. Mentaschi , E. Fischer , and K. Wyser . 2018. “Extreme Heat Waves Under 1.5 °C and 2 °C Global Warming.” Environmental Research Letters 13, no. 5: 054006. 10.1088/1748-9326/aab827.

[ele70087-bib-0023] Duncan, A. B. , S. Fellous , and O. Kaltz . 2011. “Reverse Evolution: Selection Against Costly Resistance in Disease‐Free Microcosm Populations of *Paramecium caudatum* .” Evolution 65, no. 12: 3462–3474. 10.1111/j.1558-5646.2011.01388.x. 22133218

[ele70087-bib-0024] Fire, A. , S. Xu , M. K. Montgomery , S. A. Kostas , S. E. Driver , and C. C. Mello . 1998. “Potent and Specific Genetic Interference by Double‐Stranded RNA in *Caenorhabditis elegans* .” Nature 391, no. 6669: 806–811. 10.1038/35888. 9486653

[ele70087-bib-0025] Franke, F. , N. Raifarth , J. Kurtz , and J. P. Scharsack . 2019. “Consequences of Divergent Temperature Optima in a Host–Parasite System.” Oikos 128: 869–880. 10.1111/oik.05864.

[ele70087-bib-0026] Fuxa, J. , and A. Richter . 1998. “Repeated Reversion of Resistance to Nucleopolyhedrovirus by *Anticarsia gemmatalis* .” Journal of Invertebrate Pathology 71, no. 2: 159–164. 9500947 10.1006/jipa.1997.4724

[ele70087-bib-0027] Ghildiyal, M. , and P. D. Zamore . 2009. “Small Silencing RNAs: An Expanding Universe.” Nature Reviews. Genetics 10, no. 2: 94–108. 10.1038/nrg2504. PMC2724769 19148191

[ele70087-bib-0028] Gouvêa, D. Y. , E. Z. Aprison , and I. Ruvinsky . 2015. “Experience Modulates the Reproductive Response to Heat Stress in *C. elegans* via Multiple Physiological Processes.” PLoS One 10, no. 12: e0145925. 10.1371/journal.pone.0145925. 26713620 PMC4699941

[ele70087-bib-0029] Graham, A. L. , J. E. Allen , and A. F. Read . 2005. “Evolutionary Causes and Consequences of Immunopathology.” Annual Review of Ecology, Evolution, and Systematics 36, no. 1: 373–397. 10.1146/annurev.ecolsys.36.10.

[ele70087-bib-0030] Hall, S. R. , C. R. Becker , M. A. Duffy , and C. E. Cáceres . 2010. “Variation in Resource Acquisition and Use Among Host Clones Creates Key Epidemiological Trade‐Offs.” American Naturalist 176, no. 5: 557–565. 10.1086/656523. 20887188

[ele70087-bib-0031] Hall, S. R. , C. R. Becker , M. A. Duffy , and C. E. Caceres . 2012. “A Power‐Efficiency Trade‐Off in Resource Use Alters Epidemiological Relationships.” Ecology 93, no. 3: 645–656. 22624218 10.1890/11-0984.1

[ele70087-bib-0033] Hector, T. E. , A.‐L. M. Gehman , and K. C. King . 2023. “Infection Burdens and Virulence Under Heat Stress: Ecological and Evolutionary Considerations.” Philosophical Transactions of the Royal Society of London. Series B, Biological Sciences 378: 20220018. 10.1098/rstb.2022.0018. 36744570 PMC9900716

[ele70087-bib-0034] Hector, T. E. , C. M. Sgrò , and M. D. Hall . 2021. “Thermal Limits in the Face of Infectious Disease: How Important Are Pathogens?” Global Change Biology 27, no. 19: 4469–4480. 10.1111/gcb.15761. 34170603

[ele70087-bib-0035] Hodgkin, J. , A. Coulson , and P. Kuwabara . 2001. “Mapping Useful GFP Insertions; Evidence for Local Suppression of Recombination.” Worm Breeder's Gazette 16, no. 5: 20.

[ele70087-bib-0036] Hodgkin, J. , M. A. Félix , L. C. Clark , D. Stroud , and M. J. Gravato‐Nobre . 2013. “Two Leucobacter Strains Exert Complementary Virulence on Caenorhabditis Including Death by Worm‐Star Formation.” Current Biology 23, no. 21: 2157–2161. 10.1016/j.cub.2013.08.060. 24206844 PMC3898767

[ele70087-bib-0037] IPCC . 2013. “Climate Change 2013: The Physical Science Basis.” In Contribution of Working Group I to the Fifth Assessment Report of the Intergivernmental Panel on Climate Change, 1535. Cambridge University Press.

[ele70087-bib-0038] Janda, M. , L. Lamparová , A. Zubíková , L. Burketová , J. Martinec , and Z. Krčková . 2019. “Temporary Heat Stress Suppresses PAMP‐Triggered Immunity and Resistance to Bacteria in *Arabidopsis thaliana* .” Molecular Plant Pathology 20, no. 7: 1005–1012. 10.1111/mpp.12799. 30924595 PMC6589723

[ele70087-bib-0039] Johnson, P. T. J. , R. S. Ostfeld , and F. Keesing . 2015. “Frontiers in Research on Biodiversity and Disease.” Ecology Letters 18, no. 10: 1119–1133. 10.1111/ele.12479. 26261049 PMC4860816

[ele70087-bib-0040] Johnson, P. T. J. , T. E. Stewart Merrill , A. D. Dean , and A. Fenton . 2024. “Diverging Effects of Host Density and Richness Across Biological Scales Drive Diversity‐Disease Outcomes.” Nature Communications 15, no. 1: 1937. 10.1038/s41467-024-46091-4. PMC10908850 38431719

[ele70087-bib-0041] Jones, K. E. , N. G. Patel , M. A. Levy , et al. 2008. “Global Trends in Emerging Infectious Diseases.” Nature 451, no. 7181: 990–993. 10.1038/nature06536. 18288193 PMC5960580

[ele70087-bib-0042] Keesing, F. , R. D. Holt , and R. S. Ostfeld . 2006. “Effects of Species Diversity on Disease Risk.” Ecology Letters 9, no. 4: 485–498. 10.1111/j.1461-0248.2006.00885.x. 16623733

[ele70087-bib-0043] Keesing, F. , and R. S. Ostfeld . 2021. “Dilution Effects in Disease Ecology.” Ecology Letters 24, no. 11: 2490–2505. 10.1111/ele.13875. 34482609 PMC9291114

[ele70087-bib-0044] Kimes, N. E. , C. J. Grim , W. R. Johnson , et al. 2012. “Temperature Regulation of Virulence Factors in the Pathogen *Vibrio coralliilyticus* .” ISME Journal 6, no. 4: 835–846. 10.1038/ismej.2011.154. 22158392 PMC3309362

[ele70087-bib-0045] Kingsolver, J. G. , H. Arthur Woods , L. B. Buckley , K. A. Potter , H. J. MacLean , and J. K. Higgins . 2011. “Complex Life Cycles and the Responses of Insects to Climate Change.” Integrative and Comparative Biology 51, no. 5: 719–732. 10.1093/icb/icr015. 21724617

[ele70087-bib-0046] Koskella, B. 2018. “Resistance Gained, Resistance Lost: An Explanation for Host‐Parasite Coexistence.” PLoS Biology 16, no. 9: e3000013. 10.1371/journal.pbio.3000013. 30248103 PMC6171958

[ele70087-bib-0047] Kraaijeveld, A. R. , and H. C. J. Godfray . 1997. “Trade‐Off Between Parasitoid Resistance and Larval Competitive Ability in *Drosophila melanogaster* .” Nature 389, no. 6648: 278–280. 10.1038/38483. 9305840

[ele70087-bib-0048] Kunze, C. , P. Luijckx , A. L. Jackson , and I. Donohue . 2022. “Alternate Patterns of Temperature Variation Bring About Very Different Disease Outcomes at Different Mean Temperatures.” eLife 11: e72861. 10.7554/eLife.72861. 35164901 PMC8846586

[ele70087-bib-0049] Lafferty, K. D. , and E. A. Mordecai . 2016. “The Rise and Fall of Infectious Disease in a Warmer World.” F1000Research 5: 2040. 10.12688/f1000research.8766.1. PMC4995683 27610227

[ele70087-bib-0050] Lee, J. H. , H. S. Yun , and C. Kwon . 2012. “Molecular Communications Between Plant Heat Shock Responses and Disease Resistance.” Molecules and Cells 34, no. 2: 109–116. 10.1007/s10059-012-0121-3. 22710621 PMC3887810

[ele70087-bib-0051] Legüe, M. , M. Caneo , B. Aguila , B. Pollak , and A. Calixto . 2022. “Interspecies Effectors of a Transgenerational Memory of Bacterial Infection in *Caenorhabditis elegans* .” iScience 25, no. 7: 104627. 10.1016/j.isci.2022.104627. 35800768 PMC9254006

[ele70087-bib-0052] Li, J. , N. Guttmann , G. C. Drew , T. E. Hector , J. Wolinska , and K. C. King . 2024a. “Excess Mortality of Infected Ectotherms Induced by Warming Depends on Pathogen Kingdom and Evolutionary History.” PLoS Biology 22, no. 11: e3002900. 10.1371/journal.pbio.3002900. 39556605 PMC11611255

[ele70087-bib-0053] Li, J. D. , Y. Y. Gao , E. J. Stevens , and K. C. King . 2024b. “Dual Stressors of Infection and Warming Can Destabilize Host Microbiomes.” Philosophical Transactions of the Royal Society of London. Series B, Biological Sciences 379, no. 1901: 20230069. 10.1098/rstb.2023.0069. 38497264 PMC10945407

[ele70087-bib-0056] Liew, P. K. , I. Zulkifli , M. Hair‐Bejo , A. R. Omar , and D. A. Israf . 2003. “Effects of Early Age Feed Restriction and Heat Conditioning on Heat Shock Protein 70 Expression, Resistance to Infectious Bursal Disease, and Growth in Male Broiler Chickens Subjected to Heat Stress.” Poultry Science 82, no. 12: 1879–1885. 10.1093/ps/82.12.1879. 14717545

[ele70087-bib-0057] Liu, X. , S. Lyu , S. Zhou , and C. J. A. Bradshaw . 2016. “Warming and Fertilization Alter the Dilution Effect of Host Diversity on Disease Severity.” Ecology 97: 1680–1689. 10.1890/15-1784.1. 27859159

[ele70087-bib-0059] McIntirea, K. M. , M. K. Dziuba , E. B. Haywood , et al. 2024. “Transgenerational Virulence: Maternal Pathogen Exposure Reduces Offspring Fitness.” *bioRxiv*. 10.1101/2023.03.14.532659. PMC12411831 40908793

[ele70087-bib-0060] Mikonranta, L. , J. Mappes , J. Laakso , and T. Ketola . 2015. “Within‐Host Evolution Decreases Virulence in an Opportunistic Bacterial Pathogen.” BMC Evolutionary Biology 15, no. 1: 165. 10.1186/s12862-015-0447-5. 26282271 PMC4539714

[ele70087-bib-0061] Moore, R. S. , R. Kaletsky , and C. T. Murphy . 2019. “Piwi/PRG‐1 Argonaute and TGF‐β Mediate Transgenerational Learned Pathogenic Avoidance.” Cell 177, no. 7: 1827–1841. 10.1016/j.cell.2019.05.024. 31178117 PMC7518193

[ele70087-bib-0062] Mora, C. , T. McKenzie , I. M. Gaw , et al. 2022. “Over Half of Known Human Pathogenic Diseases Can Be Aggravated by Climate Change.” Nature Climate Change 12: 869–875. 10.1038/s41558-022-01426-1. PMC9362357 35968032

[ele70087-bib-0064] van der Most, P. J. , B. de Jong , H. K. Parmentier , and S. Verhulst . 2011. “Trade‐Off Between Growth and Immune Function: A Meta‐Analysis of Selection Experiments.” Functional Ecology 25, no. 1: 74–80. 10.1111/j.1365-2435.2010.01800.x.

[ele70087-bib-0065] Oh, M. H. , S. M. Lee , D. H. Lee , and S. H. Choi . 2009. “Regulation of the *Vibrio vulnificus* hupA Gene by Temperature Alteration and Cyclic AMP Receptor Protein and Evaluation of Its Role in Virulence.” Infection and Immunity 77, no. 3: 1208–1215. 10.1128/IAI.01006-08. 19139193 PMC2643628

[ele70087-bib-0066] O'Rourke, D. , M. J. Gravato‐Nobre , D. Stroud , et al. 2023. “Isolation and Molecular Identification of Nematode Surface Resistants With Resistance to Bacterial Pathogens.” G3 Genes Genomes Genetics 13, no. 5: jkad056. 10.1093/g3journal/jkad056. 36911920 PMC10151413

[ele70087-bib-0067] Palominos, M. F. , L. Verdugo , C. Gabaldon , et al. 2017. “Transgenerational Diapause as an Avoidance Strategy Against Bacterial Pathogens in *Caenorhabditis elegans* .” mBio 8, no. 5: e01234‐17. 10.1128/mBio.01234-17. 29018118 PMC5635688

[ele70087-bib-0068] Penley, M. J. , A. B. Greenberg , A. Khalid , S. R. Namburar , and L. T. Morran . 2018. “No Measurable Fitness Cost to Experimentally Evolved Host Defence in the *Caenorhabditis elegans* ‐ *Serratia marcescens* Host‐Parasite System.” Journal of Evolutionary Biology 31, no. 12: 1976–1981. 10.1111/jeb.13372. 30187979

[ele70087-bib-0069] Prithika, U. , V. Deepa , and K. Balamurugan . 2016. “External Induction of Heat Shock Stimulates the Immune Response and Longevity of *Caenorhabditis elegans* Towards Pathogen Exposure.” Innate Immunity 22, no. 6: 466–478. 10.1177/1753425916654557. 27317398

[ele70087-bib-0072] Rohr, J. R. , T. R. Raffel , A. R. Blaustein , P. T. Johnson , S. H. Paull , and S. Young . 2013. “Using Physiology to Understand Climate‐Driven Changes in Disease and Their Implications for Conservation.” Conservation Physiology 1, no. 1: cot022. 10.1093/conphys/cot022. 27293606 PMC4732440

[ele70087-bib-0074] Schmid‐Hempel, P. 2003. “Variation in Immune Defence as a Question of Evolutionary Ecology.” Proceedings of the Royal Society of London. Series B: Biological Sciences 270, no. 1513: 357–366. 10.1098/rspb.2002.2265. PMC1691258 12639314

[ele70087-bib-0075] Schneider, D. , and J. Ayres . 2008. “Two Ways to Survive Infection: What Resistance and Tolerance Can Teach Us About Treating Infectious Diseases.” Nature Reviews. Immunology 8: 889–895. 10.1038/nri2432. PMC4368196 18927577

[ele70087-bib-0076] Schwenke, R. A. , B. P. Lazzaro , and M. F. Wolfner . 2016. “Reproduction–Immunity Trade‐Offs in Insects.” Annual Review of Entomology 61, no. 1: 239–256. 10.1146/annurev-ento-010715-023924. PMC5231921 26667271

[ele70087-bib-0077] Sekajova, Z. , E. Rosa , F. Spagopoulou , P.‐I. Zervakis , and M. I. Lind . 2022. “Temperature‐Induced Compensatory Growth in the Nematode *Caenorhabditis elegans* Is Regulated by a Thermosensitive TRP Channel and Influences Reproductive Rate.” Functional Ecology 36: 2176–2187. 10.1111/1365-2435.14116.

[ele70087-bib-0080] Shocket, M. S. , D. Vergara , A. J. Sickbert , et al. 2018. “Parasite Rearing and Infection Temperatures Jointly Influence Disease Transmission and Shape Seasonality of Epidemics.” Ecology 99: 1975–1987. 10.1002/ecy.2430. 29920661

[ele70087-bib-0081] Singh, V. , and A. Aballay . 2006. “Heat Shock and Genetic Activation of HSF‐1 Enhance Immunity to Bacteria.” Cell Cycle 5, no. 21: 2443–2446. 10.4161/cc.5.21.3434. 17106259

[ele70087-bib-0083] Strauss, A. T. , J. L. Hite , M. S. Shocket , C. E. Cáceres , M. A. Duffy , and S. R. Hall . 2017. “Rapid Evolution Rescues Hosts From Competition and Disease But—Despite a Dilution Effect—Increases the Density of Infected Hosts.” Proceedings of the Royal Society B 284, no. 1868: 20171970. 29212726 10.1098/rspb.2017.1970 PMC5740282

[ele70087-bib-0085] Walsman, J. C. , A. T. Strauss , J. L. Hite , M. S. Shocket , and S. R. Hall . 2023. “A Paradox of Parasite Resistance: Disease‐Driven Trophic Cascades Increase the Cost of Resistance, Selecting for Lower Resistance With Parasites Than Without Them.” Evolutionary Ecology 37, no. 1: 53–74. 10.1007/s10682-022-10203-7.

[ele70087-bib-0086] Webster, J. , and M. Woolhouse . 1999. “Cost of Resistance: Relationship Between Reduced Fertility and Increased Resistance in a Snail–Schistosome Host–Parasite System.” Proceedings of the Royal Society of London ‐ Series B: Biological Sciences 266, no. 1417: 391–396. 10.1098/rspb.1999.0650.

[ele70087-bib-0087] Wendling, C. C. , J. Lange , H. Liesegang , et al. 2022. “Higher Phage Virulence Accelerates the Evolution of Host Resistance.” Proceedings. Biological Sciences 289, no. 1984: 20221070. 10.1098/rspb.2022.1070. 36196537 PMC9532999

[ele70087-bib-0088] Wibisono, P. , and J. Sun . 2023. “Pathogen Infection Induces Specific Transgenerational Modifications to Gene Expression and Fitness in *Caenorhabditis elegans* .” Frontiers in Physiology 14: 1225858. 10.3389/fphys.2023.1225858. 37811492 PMC10556243

[ele70087-bib-0089] Wolinska, J. , and K. C. King . 2009. “Environment Can Alter Selection in Host‐Parasite Interactions.” Trends in Parasitology 25, no. 5: 236–244. 10.1016/j.pt.2009.02.004. 19356982

[ele70087-bib-0090] Wudu, K. , A. Abegaz , L. Ayele , and M. Ybabe . 2023. “The Impacts of Climate Change on Biodiversity Loss and Its Remedial Measures Using Nature Based Conservation Approach: A Global Perspective.” Biodiversity and Conservation 32: 3681–3701. 10.1007/s10531-023-02656-1.

[ele70087-bib-0091] Xiao, R. , B. Zhang , Y. Dong , et al. 2013. “A Genetic Program Promotes *C. elegans* Longevity at Cold Temperatures via a Thermosensitive TRP Channel.” Cell 152, no. 4: 806–817. 10.1016/j.cell.2013.01.020. 23415228 PMC3594097

[ele70087-bib-0092] Zhang, W. , V. H. W. Rudolf , and C. S. Ma . 2015. “Stage‐Specific Heat Effects: Timing and Duration of Heat Waves Alter Demographic Rates of a Global Insect Pest.” Oecologia 179: 947–957. 10.1007/s00442-015-3409-0. 26255274

