# Supplementary material for: Warming During Different Life Stages has Distinct Impacts on Host Resistance Ecology and Evolution
Source: Ecol Lett. 2025 Feb 21;28(2):e70087. doi: 10.1111/ele.70087 (PMC11843851; doi:10.1111/ele.70087)
Supplement: Supplementary file 1 — Appendix S1. [file ELE-28-0-s001.docx]

**Supplementary material**

| Table 1. Predictive framework for host resistance evolution.   \| **Developmental**  **temperature** \| **Adult-stage temperature** \| **Pathogen**  **Presence** \| **Ecological response shaping selection** \| **Prediction** \| \| --- \| --- \| --- \| --- \| --- \| \| 20°C \| 20°C \| No \| high relative cost for resistant genotype \| loss of genetic-based resistance \| \| 20°C \| 25°C \| No \| high relative cost for resistant genotype \| loss of genetic-based resistance \| \| 25°C \| 20°C \| No \| 1. similar fecundity for both genotypes 2. lowest fecundity among all temperature regimes for both genotypes \| co-existence \| \| 25°C \| 25°C \| No \| similar fecundity for both genotypes \| co-existence \| \| 20°C \| 20°C \| Yes \| 1. high relative cost for resistant genotype 2. dilution effect \| loss of genetic-based resistance \| \| 20°C \| 25°C \| Yes \| 1. high relative cost for resistant genotype 2. dilution effect 3. increased pathogen virulence \| coexistence \| \| 25°C \| 20°C \| Yes \| 1. low relative cost for resistant genotype 2. plastic defense for susceptible genotype \| coexistence \| \| 25°C \| 25°C \| Yes \| 1. plastic defense for susceptible genotype 2. increased pathogen virulence \| spread of genetic-based resistance \|    |
| --- | --- | --- | --- | --- | --- | --- | --- | --- | --- | --- | --- | --- | --- | --- | --- | --- | --- | --- | --- | --- | --- | --- | --- | --- | --- | --- | --- | --- | --- | --- | --- | --- | --- | --- | --- | --- | --- | --- | --- | --- | --- | --- | --- | --- | --- |
| Fig. S1. Loss and fixation of resistance across treatments and host generations. A. Loss of resistance. B. Fixation of resistance. X axis represents host generations, y axis represents number of replicate populations. |
|  |
| Fig. S2. Mean relative fitness of resistant genotype for 1-5 and 6-10 host generations. A. Mean relative fitness of resistant genotype for 1-5 generations. B. Mean relative fitness of resistant genotype for 6-10 generations. In each facetted plot, the diamond shape represents mean, error bar represents mean$\pm$SD, each point represents relative fitness of resistant genotype in one replicate.   \|  \| \| --- \| \| Fig. S3. Genotype frequency dynamics for 10 host generations across infection and temperature regimes, after excluding replicates where worms were added from stock plates to achieve the fixed population size. Host populations were started at 50:50 ratio of the two genotypes. \| |

## **The mechanistic model**

## **Methods**

Here we describe the mechanistic model which we use primarily to determine if the dilution of pathogen by a less competent (resistant) genotype can disproportionately benefit a susceptible genotype. We define four types of host individual: an uninfected susceptible genotype (with density denoted by $U_{s}$); an infected susceptible genotype ($I_{s}$); an uninfected resistant genotype ($U_{r}$); and an infected resistant genotype ($I_{r}$). These hosts interact with the environment, which contains a free-living pathogen, with density denoted by $P$. Hosts replicate with an intrinsic rate of $\lambda_{s}$ and $\lambda_{r}$, but suffer a loss in fecundity of size $\varphi_{s}$ and $\varphi_{r}$ (between zero and one) for the susceptible and resistant genotypes respectively. For simplicity, we will assume that both host genotypes have the same natural mortality rate of $\theta$, and that they share the same food resources in the environment which can support a maximum density of $q$.

We will assume two different modes of transmission. Both genotypes can be infected by free-living pathogen in the environment with a rate per unit pathogen density of $\beta_{E}$, and the susceptible genotype is able to transmit to other susceptible hosts horizontally with a rate per unit host of $\beta_{H}$. We assume that the resistant genotype is resistant to pathogen attachment, so that it cannot be infected horizontally. Finally, we assume that the susceptible genotype has a mortality rate of $\sigma_{s}$, while there is evidence to suggest that the resistant genotype does not suffer a virulence cost of mortality.

The free-living pathogen undergoes several processes. They will replicate with an intrinsic growth rate of $\tilde{\rho}$ and decays at a rate of $\tilde{\delta}$, and the environment is assumed to be able to support a total density of $\tilde{K}$. When an infection event occurs, a density $\tilde{\eta}$ of pathogen per host is removed and infected hosts shed a density $\tilde{a}$ of pathogen per host per unit time. The resistant genotype sheds much less pathogens and is much less exploited, to capture this dilution effect, we will assume that all resistant hosts remove a density $\tilde{\gamma}$ of pathogen per unit time. The removal of pathogen could benefit both genotypes. The ordinary differential equations (ODEs) for these assumptions are shown below in equations (1)-(5), and a schematic for the modelling framework can be found in Fig. S4:

| $\frac{dU_{s}}{dt}=\lambda_{s}\left( U_{s}+\left( 1-\varphi_{s} \right)I_{s} \right)\left( 1-\frac{N}{q} \right)-\theta U_{s}-\beta_{E}U_{s}P-\beta_{H}U_{s}I_{s},$ | (S1) |
| --- | --- |
| $\frac{dI_{s}}{dt}=\beta_{E}U_{s}P+\beta_{H}U_{s}I_{s}-\theta I_{s}-\sigma_{s}I_{s},$ | (S2) |
| $\frac{dU_{r}}{dt}=\lambda_{r}\left( U_{r}+\left( 1-\varphi_{r} \right)I_{r} \right)\left( 1-\frac{N}{q} \right)-\theta U_{r}-\beta_{E}U_{r}P,$ | (S3) |
| $\frac{dI_{s}}{dt}=\beta_{E}U_{r}P+\beta_{H}U_{r}I_{r}-\theta I_{r},$ | (S4) |
| $\frac{dP}{dt}=\tilde{\rho}P\left( 1-\frac{P}{\tilde{K}} \right)-\tilde{\delta}P-\tilde{\eta}\beta_{E}P\left( U_{s}+U_{r} \right)+\tilde{a}\left( I_{s}+I_{r} \right)-\tilde{\gamma}P\left( U_{r}+I_{r} \right),$ | (S5) |

where $N=U_{s}+I_{s}+U_{r}+I_{r}$ is the total host density.


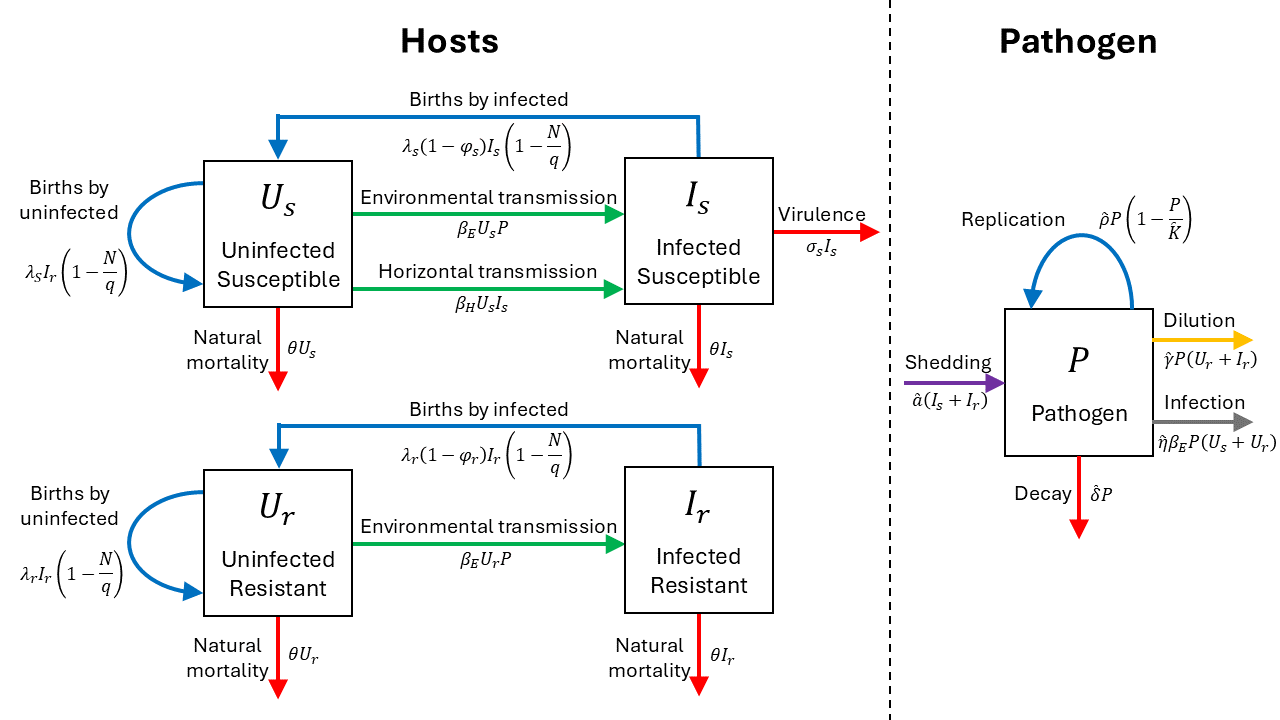


Fig. S4: Model schematic for equations (1)-(5). An arrow indicates a flow into or out of a particular state (boxes), with the number of hosts per unit time or pathogen per unit time given mathematically on the arrow, together with the interpretation. All blue arrows indicate replication events, green arrows are transmission events and red arrows are death (either hosts or pathogen). The pathogen also has three unique types (all arrows moving from left to write in purple, orange and grey).

We can reduce the number of parameters in this system by rescaling our state variables and time in the following way:

$$u_{j}=\frac{U_{j}}{q}; v_{j}=\frac{I_{j}}{q};n=\frac{N}{q};z=\frac{\beta_{E}P}{\lambda_{s}}; \tau=\lambda_{s}t,$$

for $j\in\{s,r\}$. This rescaling ensure that the host populations should sum to close to 1 at equilibrium, each unit of time is the average reproductive time of the susceptible genotype, and the pathogen is rescaled so that the typical environmental transmission rate ($\beta_{E}P$) is on the same timescale as susceptible genotype reproduction. This rescaled system is given by:

| $\frac{du_{s}}{d\tau}=\left( u_{s}+\left( 1-\varphi_{s} \right)v_{s} \right)\left( 1-n \right)-du_{s}-u_{s}z-\beta u_{s}v_{s},$ | (S6) |
| --- | --- |
| $\frac{dv_{s}}{d\tau}=u_{s}z-dv_{s}-\alpha_{s}v_{s},$ | (S7) |
| $\frac{du_{r}}{d\tau}=\lambda\left( u_{r}+\left( 1-\varphi_{r} \right)v_{r} \right)\left( 1-n \right)-du_{r}-u_{r}z,$ | (S8) |
| $\frac{dv_{r}}{d\tau}=u_{r}z-dv_{r},$ | (S9) |
| $\frac{dz}{d\tau}=\rho z\left( 1-\frac{z}{K} \right)-\delta z-\eta\left( u_{s}+u_{r} \right)z+a\left( v_{s}+v_{r} \right)-\gamma\left( u_{r}+v_{r} \right)z.$ | (S10) |

Here, we define the following scaled parameters for the host population:

| $d=\frac{\theta}{\lambda_{s}}; \alpha_{W}=\frac{\sigma_{W}}{\lambda_{s}};r=\frac{r_{M}}{\lambda_{s}};\beta=\frac{\beta_{H}q}{\lambda_{s}},$ | (S11) |
| --- | --- |

and the following for the pathogen:

| $\rho=\frac{\tilde{\rho}}{\lambda_{s}}; K=\frac{\beta_{E}\tilde{K}}{\lambda_{s}}; \delta=\frac{\tilde{\delta}}{\lambda_{s}}; \eta=\frac{\tilde{\eta}\beta_{E}q}{\lambda_{s}};a=\frac{\tilde{a}}{\lambda_{s}}; \gamma=\frac{\tilde{\gamma}q}{\lambda_{s}}.$ | (S12) |
| --- | --- |

For our results, we define the following metric denoted by $\chi$, which is a measure of the ratio between susceptible, $x_{s}=u_{s}+v_{s},$ and resistant, $x_{r}=u_{r}+v_{r}$ genotypes:

| $\chi=\frac{x_{s}-x_{r}}{x_{s}+x_{r}}.$ | (S13) |
| --- | --- |

This measure will take a positive value if the susceptible genotype is dominating, capping at $\chi=1$ which occurs when the susceptible genotype fully excludes the resistant genotype, with a similar explanation for the resistant genotype and negative values.

## **Simulation**

All results from the mathematical model will utilise random parameterisation. This is for two reasons: firstly it allows us to conduct a sensitivity analysis and determine which parameters are most important for the response variable $\chi$; and secondly because we don’t have accurate estimates for what these parameters should be. We will use the rescaled system (S6)-(S10) to conduct our analyses.

We will fix most of the pathogen parameters to be $\rho=1$, $K=1$, $\delta=0.01$, $a=0.001$ and $\eta=0.0001$ and then we will randomise all other parameters in the following way:

$$\varphi_{s}\sim Uniform\left( 0, 1 \right),$$

$$\varphi_{r}\sim Uniform\left( 0, 1 \right),$$

$$\alpha_{s}\sim Uniform\left( 0, 0.5 \right),$$

$$\gamma\sim Uniform\left( 0, 20 \right),$$

$$d \sim Uniform\left( 0, 0.001 \right),$$

$$\beta\sim Uniform\left( 0, 2 \right).$$

To choose the value of $\lambda=\lambda_{r}/\lambda_{s}$, which is the ratio of the genotype’s intrinsic growth rates, we will initially choose a scale factor uniformly between 1 and 5 ($s \sim Uniform(1, 5)$, and then choosing $\lambda=s$ or $\lambda=1/s$ with equal probabity.

## **Results**

We now present the main findings from our modelling framework. All simulations were completed in Python 3.9.7 using Visual Studio Code, and utilise 10,000 independently parameterised systems.

### **The genotype with higher fecundity generally wins**

From panel A of Fig. S5, we can see that whenever $\log_{10} (\lambda)<0$, which occurs when the susceptible genotype has a higher intrinsic growth rate than the resistant one ($\lambda_{s}>\lambda_{r}$), there are many more instances of the susceptible genotype outcompeting the resistant genotype (many more blue dots than red dots), and vice-versa for when $\log_{10} (\lambda)>0$. In Fig. S5C, we see that there is an unequal distribution, with there being more instances of the susceptible genotype outcompeting (blue bar) compared to the resistant genotype (red bar) despite the susceptible genotype suffering from an excess in mortality on infection.


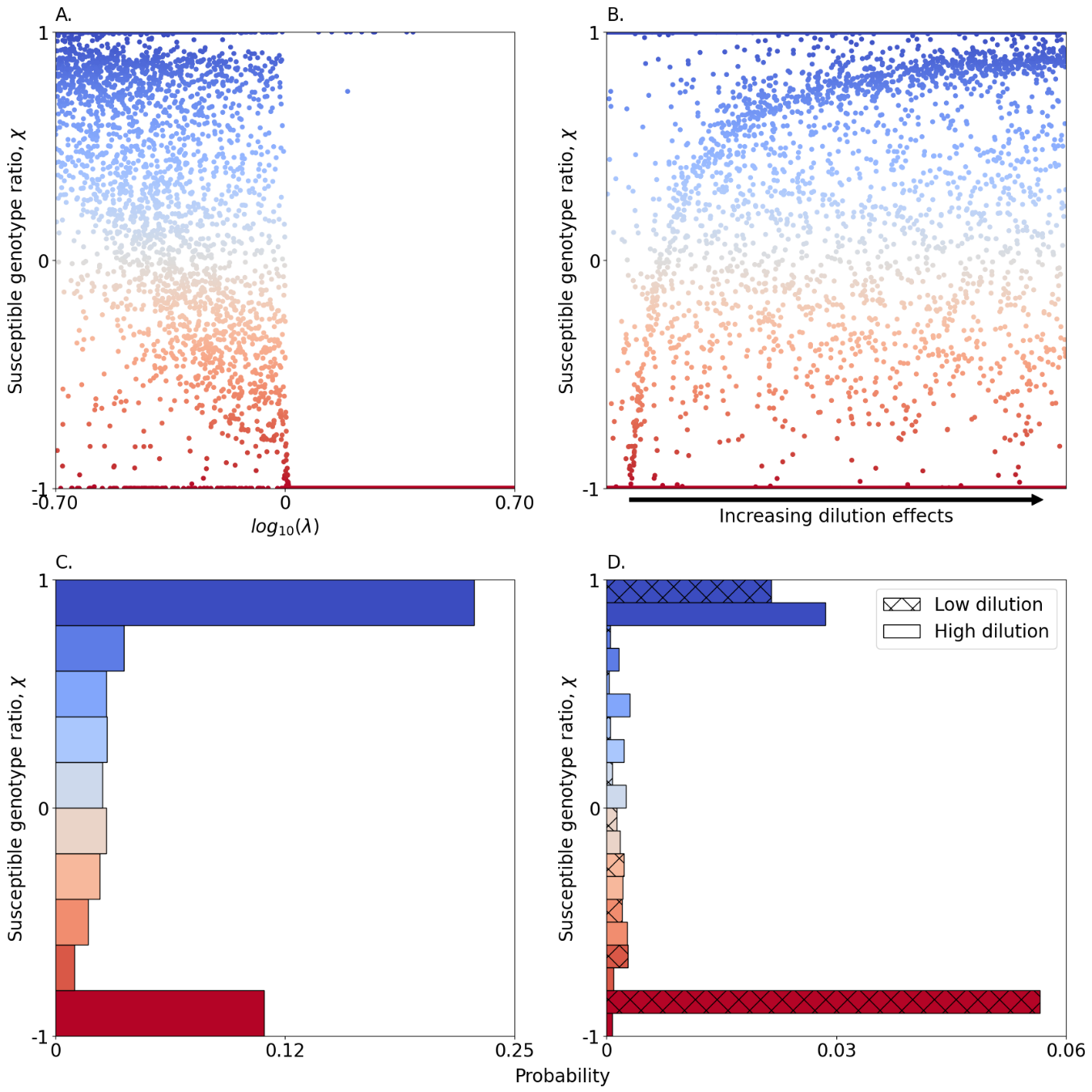


Fig. S5: Results from 10,000 randomly parameterised simulations of equations (S6)-(S10). In all figures, the y-axis denotes the susceptible genotype ratio as defined by equation (S13), with blue colours ($\chi>0$) indicating susceptible dominance, and red colours ($\chi<0$) indicating resistant dominance. A. The ratio of intrinsic fecundities is varied on a log scale, with values less than 0 indicating the susceptible genotype reproduces faster than the resistant, and vice versa for values greater than 0. B. The effect of increasing dilution effect. The x-axis is an increase of the non-dimensional dilution effect parameter $\gamma$. C. The overall probability distribution of $\chi$ values across all 10,000 simulations. D. A breakdown of the probability distribution for the low dilution effects (hashed bars) and high dilution effects (non-hashed bars). Low and high dilution effects are defined as the lower and upper 10% of the parameter range for the parameter $\gamma$.

### **Dilution effect plays an important role in selecting for the susceptible genotype**

We further investigate the impact of dilution effect on which genotype dominates. Mathematically, we include dilution effects as a reduction in pathogen caused by the resistant genotype, and is denoted by the $\tilde{\gamma}\left( U_{r}+I_{r} \right)P$ term in equation (S5) or the $\gamma\left( u_{r}+v_{r} \right)z$ term in equation (S10). These effects favour each genotype equally by removing pathogen from the environment, and do not adversely affect the resistant genotype.

We can see the effect of increasing the dilution effects in panel B of Fig. S5, with the susceptible genotype being selected more often as we increase the strength of the genotype. This information is also displayed in panel D, where we plot the probability of having particular $\chi$ values as a function of the dilution effects. Here, “low dilution” (hashed bars) would be any simulations where the parameter $\gamma$ was less than 10% of the maximum, while “high dilution” (plain bars) would be the upper 10%. We see a clear difference between the two regimes, with the resistant genotype mostly being selected for at low dilution regimes, while at higher dilution regimes, the susceptible genotype is almost exclusively selected for.

### **The strength of horizontal transmission is an indicator for the survivability of the susceptible genotype**

We have conducted a visual sensitivity analysis using the same data as in Fig. S5. Other than the ratio of fecundities ($\lambda$) and the strength of dilution effects ($\gamma$), the strength of horizontal transmission compared to environmental transmission is also important. We see in Fig. S6 that when horizontal is smaller than environmental transmission ($\beta<1$) there is a strong tendency for one of the two genotypes dominating (very few simulations with close to equal densities of both). As the horizontal transmission becomes higher than environmental, the tendency is for the resistant genotype to be more dominant than the susceptible. As the resistant genotype does not suffer from this horizontal transmission, increasing this parameter will have a disproportionate effect on the susceptible genotype. Thus, factors that contribute to increased horizontal transmission of pathogens, such as increased mobility of susceptible hosts, may select for resistance in the population.


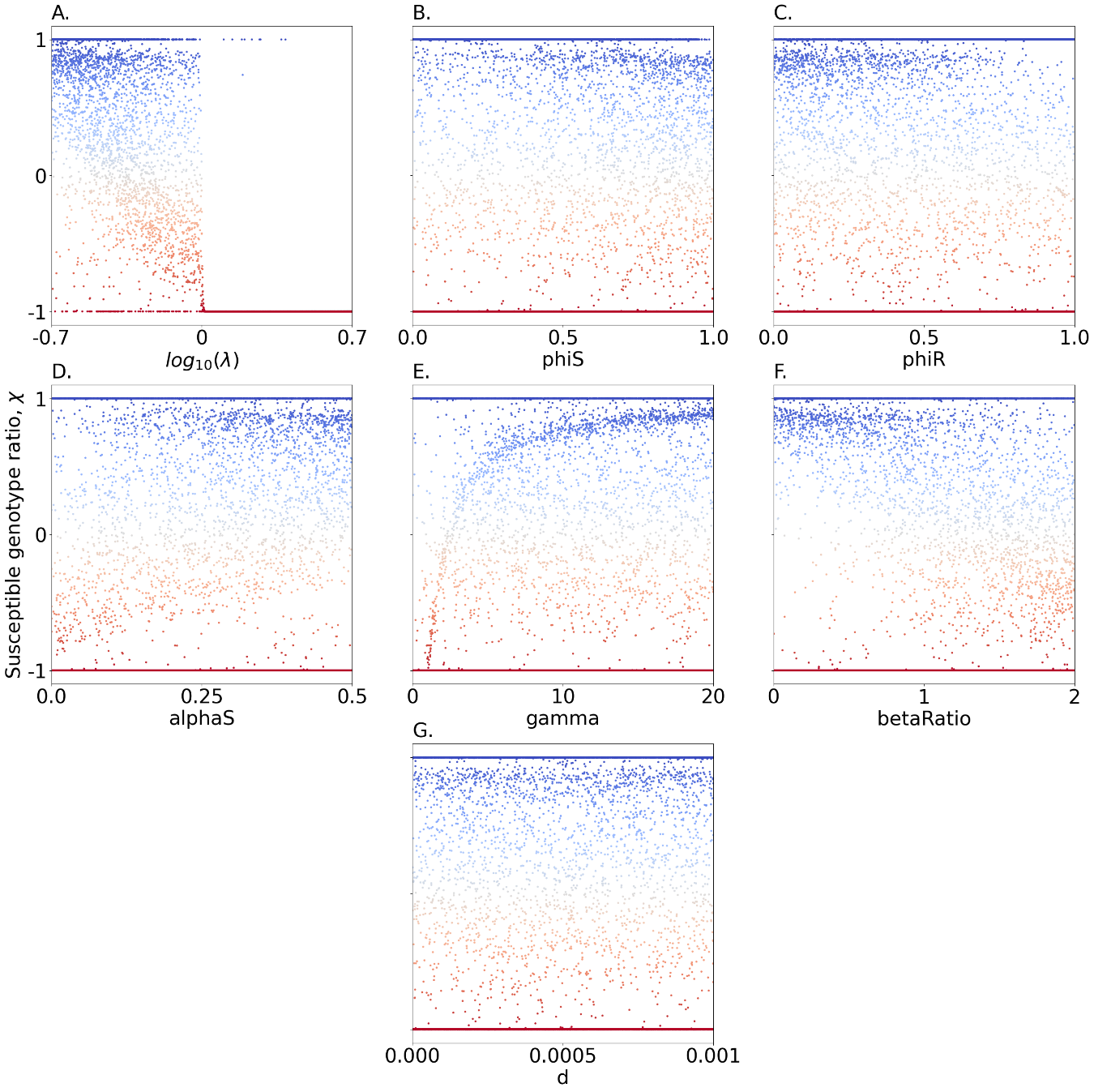


Fig. S6: Sensitivity analysis for the model (S6)-(S10) over 10,000 simulations. In all subfigures, we have the response variable being the genotype ratio $\chi$, and x axis being A. The log of the ratio of the intrinsic growth rates; B. The loss in fecundity caused by infection of the susceptible genotype; C. The loss in fecundity by infection of the resistant genotype; D. The virulence of the susceptible genotype; E. The dilution effects; F. The relative effects of horizontal transmission compared to environmental transmission; G. The natural mortality rate.
